# Node Role Selection and Rotation Scheme for Energy Efficiency in Multi-Level IoT-Based Heterogeneous Wireless Sensor Networks (HWSNs)

**DOI:** 10.3390/s24175642

**Published:** 2024-08-30

**Authors:** Tamoor Shafique, Abdel-Hamid Soliman, Anas Amjad, Lorna Uden, Debi Marie Roberts

**Affiliations:** School of Digital, Technology, Innovation and Business, Staffordshire University, Stoke-on-Trent ST4 2DE, UK; a.soliman@staffs.ac.uk (A.-H.S.); a.amjad@staffs.ac.uk (A.A.); l.uden@staffs.ac.uk (L.U.); debi.roberts@staffs.ac.uk (D.M.R.)

**Keywords:** Internet of Things, heterogeneous wireless sensor network, energy balanced routing, hierarchical routing, energy consumption rate, network lifetime, hot spot, energy holes, cluster-head rotation, relay-node rotation

## Abstract

The emergence of Internet of Things (IoT)-based heterogeneous wireless sensor network (HWSN) technology has become widespread, playing a significant role in the development of diverse human-centric applications. The role of efficient resource utilisation, particularly energy, becomes further critical in IoT-based HWSNs than it was in WSNs. Researchers have proposed numerous approaches to either increase the provisioned resources on network devices or to achieve efficient utilisation of these resources during network operations. The application of a vast proportion of such methods is either limited to homogeneous networks or to a single parameter and limited-level heterogeneity. In this work, we propose a multi-parameter and multi-level heterogeneity model along with a cluster-head rotation method that balances energy and maximizes lifetime. This method achieves up to a 57% increase in throughput to the base station, owing to improved intra-cluster communication in the IoT-based HWSN. Furthermore, for inter-cluster communication, a mathematical framework is proposed that first assesses whether the single-hop or multi-hop inter-cluster communication is more energy efficient, and then computes the region where the next energy-efficient hop should occur. Finally, a relay-role rotation method is proposed among the potential next-hop nodes. Results confirm that the proposed methods achieve 57.44%, 51.75%, and 17.63% increase in throughput of the IoT-based HWSN as compared to RLEACH, CRPFCM, and EERPMS, respectively.

## 1. Introduction

**Figure 1:**
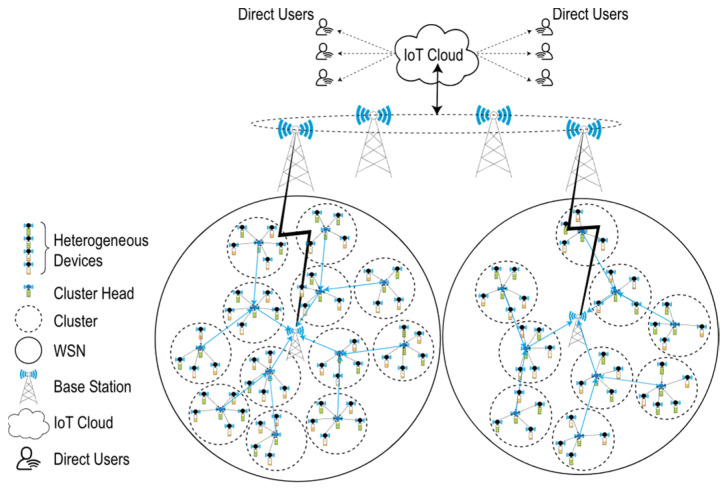
HWSN-based IoT infrastructure.

Advancements in micro-electromechanical systems (MEMS) and the emergence of the Internet of Things (IoT) enable real-time access to data, facilitating collaborative solutions to various real-world problems. The IoT enables people to experience intelligent living environments, such as smart cities [1], smart homes [2], and smart transportation [3]. These applications typically require a large number of energy-limited wireless devices deployed over extensive areas [4]. The integration of wireless sensor networks (WSNs) with IoT [5] lowers costs and improves quality of life by utilising smart sensor networks, where devices are connected to the internet [6,7]. A wireless sensor network (WSN) consists of numerous low-power microsensor nodes that work together to detect, process, and communicate information about their surrounding environment [4]. Due to their low cost, rapid deployment, and self-organisation features [8], WSNs are widely used in various applications such as marine data collection [9], pollution monitoring [10], smart farming [11], precision agriculture [11], disaster warnings [12], wildlife monitoring [13], and multi-floor building data collection [14]. Thus, integrating WSNs and IoT for effective service delivery across various applications does not require a significant paradigm shift [7]. Figure 1 illustrates the overall infrastructure of a WSN-based IoT system. Various sensors with heterogeneous attributes collect real-time environmental data, which is then communicated to a base station using clustered hierarchical routing [15]. The real-time data are made accessible through the IoT cloud, enabling coordinated service delivery in modern infrastructures like smart cities [16]. Although WSN-based IoT systems are appealing because they can operate independently in harsh environments where human presence is impractical [6], these environments also pose challenges to network longevity [17] due to the inability to replace or recharge their limited batteries [18]. Moreover, in hierarchical routing, cluster heads closer to the base station experience excessive relaying loads, leading to their premature failure [15]. Scalable and adaptable energy-efficient routing schemes are a crucial quality of service (QoS) criterion in networks with large-scale device deployment [15].

**Figure 2:**
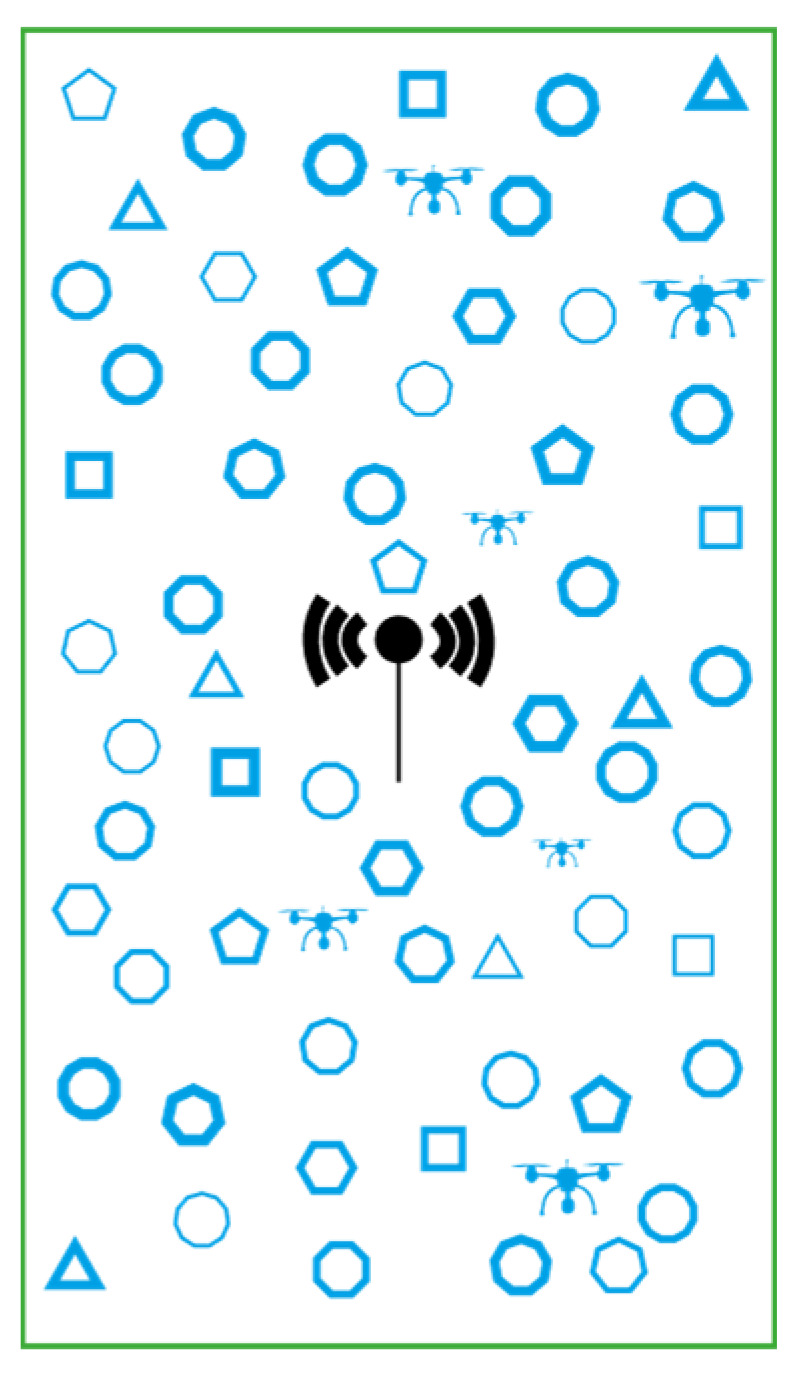
Multi-parameter and multi-level heterogeneous wireless sensor network.

Moreover, IoT-based WSNs can include sensor nodes with a wide range of capabilities, introducing a more complex variant known as IoT-based HWSN [19]. Figure 2 illustrates an m-level heterogeneous wireless sensor network, where diverse sensor nodes transmit information about the field to the base station (BS), which stores the data in a central repository. Authorised end-users with the appropriate credentials can access any required data from this repository via the internet.

The term ‘m-level’ refers to the varying functionalities and corresponding range of resources that a device can possess, such as sensing ranges, sensing tasks, mobility levels, initial energy supplies, and computational capabilities, among others [19].

Deploying sensor nodes with diverse functionalities and capabilities results in cost-effective solutions and improved performance. This makes the HWSN-based IoT a more practical alternative to traditional WSN-based IoT systems. Due to the variety of node capabilities, HWSNs can be classified into several principal categories based on differences in energy levels, computing power, connection quality, data generation rates, and node mobility.

In the category of energy heterogeneity, nodes possess varying initial energy levels or are equipped with sensors that support replaceable batteries. In terms of computational capabilities, some nodes have superior processing power and larger storage capacities than others. Regarding link heterogeneity, certain nodes provide longer-distance and more reliable communication links within the network. For data rate heterogeneity, nodes differ in their sensing schedules and the size of the data packets they generate. Finally, with respect to mobility, nodes may either be stationary or capable of movement, with mobile nodes exhibiting different speeds.

Energy heterogeneity is considered fundamental, with link, computation, and data rate heterogeneities being viewed as functions of energy heterogeneity. Additionally, mobility heterogeneity not only introduces energy challenges but also adds configuration complexities within the network [19]. This work focuses on enhancing energy efficiency within the network layer by introducing a method that ensures key requirements such as self-organisation and energy-efficient data communication. This is accomplished through dynamic clustering and routing in a network that is heterogeneous across multiple parameters and levels.

Clustering protocols are a key method for energy-efficient data collection, organising nodes into clusters. Each cluster is led by a cluster head (CH), which gathers data from its members and sends it to an external base station (BS), often through multi-hop communication between cluster heads. The process typically involves two main phases: (i) the election of cluster heads and the formation of clusters, where nodes elect cluster heads and join a cluster, and (ii) the operational phase, where data are transmitted to a centralised BS. Clustering ensures that only nodes with the cluster-head role endure the overhead associated with multi-hop communication in long-distance transmission. To ensure energy is used efficiently, the process of electing cluster heads and forming clusters may be periodically repeated.

The strategy of rotating the cluster-head role [6,8,17,20,21,22,23] in conjunction with clustering plays a crucial role in extending the network’s lifespan. This approach minimises the need for significant traffic during leader election and cluster setup, as outgoing cluster heads directly appoint their successors. It assumes the existence of pre-established clusters, which can be formed using either static [24,25,26] or dynamic methods [21,27,28,29,30,31,32].

In current clustering protocols, cluster heads (CHs) take on more responsibilities compared to cluster members, leading to significantly higher energy consumption than their cluster member counterparts. Moreover, existing approaches do not fully account for the diverse heterogeneity of sensor nodes. In large-scale networks, cluster heads use multi-hop inter-cluster routing to transmit data to the base station, which causes cluster heads closer to the base station to encounter excessive relaying loads, resulting in their premature failure [16]. Therefore, beyond cluster-head selection and rotation to balance intra-cluster energy consumption, an optimal relay-node selection and rotation scheme is also necessary. The proposed energy-efficient routing for IoT-based multi-level heterogeneous networks aims to be adaptable and meet the requirements of networks of varying sizes, from small- to large-scale. The main contributions of this study are summarised as follows:

1. A novel approach is proposed to extend network lifetime and enhance energy efficiency in intra-cluster communication. This includes the development of a versatile heterogeneity model for multi-parameter heterogeneous networks. Additionally, a mathematical model is developed and integrated into a new cluster-head rotation algorithm, optimising and balancing energy consumption. These contributions ensure stable, energy-efficient network performance and sustainable operations.
2. A novel mathematical model is developed to dynamically identify the optimal region for relay selection, allowing any node within this region to be chosen as a relay based on resource distribution and energy costs. An algorithm is developed that incorporates the dynamic selection of relay nodes to effectively balance inter-cluster energy consumption. The proposed method significantly improves energy efficiency, by preventing premature node failure, and ensures long-term network performance.
3. A novel algorithm is developed to implement dynamic relay-role rotation among nodes within the identified optimal region. The proposed role rotation prevents excessive energy depletion in any single node and optimises inter-cluster communication. By distributing the communication load more evenly, the algorithm significantly extends the network’s lifespan and stability.

The remainder of this paper is structured as follows: Section 2 provides a critical review of the related literature. Section 3 describes the network and energy models, utilised for the analysis and evaluation of the proposed methods. Section 4 details the proposed schemes, including corresponding mathematical models, flow charts, and pseudo codes. Section 5 outlines the results and discussion. Finally, conclusions and future research directions are outlined in Section 6.

## 2. Related Work

Clustering and routing approaches have consistently demonstrated their significance in conventional wireless sensor networks (WSNs), particularly due to their contributions to scalability and energy efficiency [20,33,34,35]. While these approaches have been well studied in conventional WSNs, they can also be effectively utilised in IoT-based heterogeneous wireless sensor networks (HWSNs) to achieve energy efficiency in complex and diverse network environments [36,37,38,39,40,41]. Numerous protocols, such as SEP (stable election protocol) [30], DEEC (distributed energy efficient clustering) [31], and their derivatives like D-DEEC [42], E-DEEC [43], ED-DEEC [44], DRE-SEP [45], DARE-SEP [36], and DE-SEP [37], have been developed based on the foundational principles established by LEACH [27]. These protocols employ strategies such as randomised cluster-head rotation to evenly distribute workload, data aggregation to conserve energy, and local coordination to maintain scalability and robustness, particularly in dynamic environments.

SEP (stable election protocol), proposed by Smaragdakis et al. [30], is a LEACH-inspired clustering method that addresses two-tier energy heterogeneity by differentiating between normal and advanced nodes, the latter having greater resources. While SEP enhances the management of energy variance and introduces specific operational epochs for each node type, it faces limitations such as the necessity for frequent cluster reformation in each round and its restriction to only two levels of energy heterogeneity.

DEEC [31], which introduces a probabilistic cluster-head selection mechanism based on residual and average network energy, attempts to distribute the energy load more effectively. However, it inadvertently penalises high-energy nodes by depleting their resources at the same rate as lower-energy nodes, which can lead to suboptimal energy distribution and shortened network lifespan. Subsequent enhancements like D-DEEC [42] and E-DEEC [43] aim to address these issues by incorporating multiple levels of energy heterogeneity. Nonetheless, these protocols often fall short in environments where node capabilities vary significantly, as they tend to treat advanced nodes similarly once their energy levels converge, thus failing to fully exploit the heterogeneity within the network. Further, protocols such as ED-DEEC [44] and Qureshi et al.’s four-tier model [46] expand on these ideas by introducing more granular classifications of node energy levels and tailored probabilistic equations for cluster-head selection. However, these methods still face challenges in maintaining efficiency as node energy levels deplete, often leading to a homogenisation of nodes that undermines the benefits of the initial heterogeneity.

Mittal and Singh’s DRE-SEP [45], an enhancement of SEP [30], addresses three levels of energy heterogeneity, particularly in event-driven applications, by using a weighted probabilistic formula for cluster-head selection that factors in node distance, energy levels, and type-specific epochs. It also facilitates dual-hop communication to reduce energy consumption. Similarly, (distance-aware residual energy efficient stable election protocol) DARE-SEP [36] and (distance and distance and energy aware) DE-SEP [37] refine the SEP approach by incorporating node distance and energy levels into cluster-head selection, with DE-SEP limiting the number of cluster heads to optimise energy efficiency. However, these protocols remain primarily focused on energy heterogeneity, often neglecting other important factors such as computational capabilities, link quality, and data generation rates—key considerations in large-scale, heterogeneous IoT environments.

Another key challenge in existing approaches is the management of network heterogeneity beyond energy levels. Protocols like the energy-driven unequal clustering (EDUC) [47] and adaptations of HEED [29], such as those proposed by Chand et al. [48] and Singh et al. [49], have sought to address heterogeneities in computational power, communication link quality, and node mobility. Moreover, Singh et al. also developed an energy efficient heterogeneous DEEC protocol [50], which introduces three-level heterogeneity. However, these protocols frequently encounter issues such as inefficient load distribution, increased overhead due to frequent cluster reformation, and vulnerability to data loss when cluster-head communication is interrupted.

The introduction of more sophisticated protocols, such as multi-level HEED (ML-HEED) [51], which supports up to six levels of energy heterogeneity, demonstrates the potential for improved network lifespan and energy usage. However, the frequent need for cluster reformation at the start of each round consumes significant energy, which could otherwise be allocated to more critical network operations.

Recent protocols like the improved energy efficient clustering protocol (IEECP) by Hassan et al. [6] and hybrid routing approaches proposed by Priyadarshi et al. [38] and Naeem et al. [36] focus on prolonging network lifetime through optimised cluster formation and the use of relay nodes for long-distance communication. While these methods represent valuable progress, they are often limited by their focus on homogeneous networks or their application to only two levels of energy heterogeneity, restricting their effectiveness in more complex IoT-based HWSNs.

The authors in [39] introduced a hybrid routing approach to enhance energy efficiency in sensor networks, encompassing both normal and advanced nodes. This method involves segmenting the network into smaller regions and deploying relay nodes to optimise long-distance communication energy. Routing is performed between nodes and the base station, cluster heads, relays, and relays to the base station. However, the scheme restricts advanced and normal nodes to their predefined regions, limiting its applicability and only considering two levels of energy heterogeneity.

Moreover, innovations like the threshold-oriented and energy-harvesting enabled multi-level stable election protocol (TEM-SEP) [52] and the non-threshold-based cluster-head rotation scheme (NCHRA) [22] are indicative of the ongoing efforts to support large-scale, heterogeneous networks. However, these protocols still primarily address energy heterogeneity and often neglect other critical parameters, such as data rate variability and computational capabilities, which are essential for efficient network management in real-life IoT deployments.

Finally, Gherbi et al. [40] and Kumar et al. [41] have proposed clustering-based protocols that attempt to refine the cluster-head selection process by considering node deployment and residual energy. While these methods contribute to prolonging network lifetime, they often introduce additional complexity or overhead, which may not be sustainable in large-scale, dynamic environments.

Enhanced hierarchical clustering node collaborative scheduling (EHCNCS) [17] also rotates the cluster-head role between nodes by constructing a CH rotation candidate table. Although EHCHCS achieves significant energy efficiency and network lifetime, its applications are limited to homogeneous sensor networks. Moreover, it compromises the complete network coverage to enhance network lifetime.

Lewandowski and Płaczek [53] proposed a method that incorporates a new CH rotating algorithm along with suppression of unnecessary data transmissions. This method exploited energy heterogeneity and only cluster-head rotation and sleep–awake scheduling was considered to enhance the network lifetime. The consideration for large-scale networks and multi-hop inter-cluster communication was not included. Therefore, balance between cluster heads due to inter-cluster communication was not thoroughly considered.

Centralised energy-efficient clustering (CEEC) [20] also includes the cluster-head rotation for energy balancing among the nodes in same cluster. Moreover, it adds size-balance cluster formation (SBCF), inter-cluster energy adjustment (ICEA), and energy-aware data forwarding (EDF) schemes. CEEC is also focused on homogeneous WSNs and does not address the complex dynamics of a real-life heterogeneous wireless sensor network-based IoTs.

Despite the advancements made by the aforementioned protocols, several outstanding research problems remain unaddressed. Many existing protocols fail to fully account for the diverse heterogeneity of sensor nodes, particularly in balancing energy consumption across both intra-cluster and inter-cluster communications. This limitation leads to premature node failures, especially in large-scale networks with varying node capabilities.

Current strategies often overlook the need for an optimal relay-node selection and rotation scheme, which is essential for maintaining network longevity in heterogeneous environments. This oversight can result in uneven energy distribution and increased communication overhead.

Moreover, most existing protocols are either tailored to small-scale networks or limited to specific levels of energy heterogeneity, making them less effective in real-world IoT applications that demand flexibility and scalability.

In summary, although significant progress has been made in developing clustering and routing protocols for energy-efficient and scalable WSNs and HWSNs, several critical challenges remain. These include the need for more comprehensive management of node heterogeneity, improved strategies for balancing energy consumption, and the development of protocols that can adapt to varying network sizes and levels of heterogeneity. This research addresses these challenges by introducing innovative methods that integrate multi-level heterogeneity into the clustering and routing processes, thereby enhancing network efficiency, scalability, and longevity.

## 3. Network and Energy Models

The network architecture and energy consumption models employed in this study are summarised as follows:

### 3.1. Network Model

The proposed method addresses a heterogeneous sensor network, with the following characteristics:

1. Deployed sensor nodes possess limited heterogeneous energy levels within a specified range.
2. All deployed sensors remain static and do not change their locations.
3. The sensors have the ability to adjust their transmission power levels.
4. Sensors regularly monitor the environment, with data rates varying based on events or sensor node types.
5. The base station remains static and does not change its location.
6. The network may contain any number of nodes, accommodating various shapes and scales, with fully heterogeneous sensor nodes.

To elaborate on the concept of multi-level heterogeneity, consider a network with n levels of energy heterogeneity and m levels of data rate variability, where ‘n’ and ‘m’ are positive integers greater than zero.

where n,m>0 & $n,m\in Z^{+}$

Consider a scenario where ‘N’ represents the total number of sensors in the network and $\mu_{1},\mu_{2},\;\mu_{3},\;\ldots ,\;\mu_{n}$ denote the proportional factors corresponding to the deployed sensors at heterogeneity levels 1,2,3,…,n for a given parameter μ. These factors satisfy the equation:

$$\mu_{1}+\mu_{2}+\mu_{3}+\ldots +\mu_{n}=1$$

Thus, the total number of sensors can be expressed as:

$$\sum_{i=1}^{n}\mu_{i}\cdot N=N$$

Consider, $o_{1},o_{2},o_{3},\ldots$ as energy multipliers for nodes of types 1,2,3, and beyond, adhering to the sequence:

$$o_{1}<o_{2}<o_{3}<o_{4}<\ldots$$

Here, $o_{1}$ is assigned the value of 0, corresponding to type-1 nodes. The initial energy and data rate of the jth type of heterogeneous node can be expressed as $E^{j}=\left(E_{0}+o_{j}\cdot E_{0}\right)$ and $T^{j}=\left(T_{0}+o_{j}\cdot T_{0}\right),$ respectively, where, $E_{0}$ and $T_{0}$ represent the initial energy and packet size of the type-1 nodes, respectively.

Thus, the total network energy, $E_{network}$ and the total data traffic in the network, $T_{network}$, are defined as:

$$E_{network}=\sum_{i=1}^{n}\mu_{i}\cdot N\cdot E^{i}\;and\;T_{network}=\sum_{i=1}^{n}\mu_{i}\cdot N\cdot T^{i}$$

If $\kappa_{i}$ represents the number of type-*i* sensor nodes, then:

$$\kappa_{i}=\mu_{i}\cdot N$$

and

$$\sum_{i=1}^{n}\kappa_{i}=N$$

Thus, the total network energy, $E_{network},$ can be written as:

$$E_{network}=\kappa_{1}\cdot E^{1}+\kappa_{2}\cdot E^{2}+\ldots +\kappa_{n}\cdot E^{n}$$

$$E_{network}=\kappa_{1}\cdot \left(E_{0}+o_{1}\cdot E_{0}\right)+\kappa_{2}\cdot \left(E_{0}+o_{2}\cdot E_{0}\right)+\kappa_{3}\cdot \left(E_{0}+o_{3}\cdot E_{0}\right)+\ldots +\kappa_{n}\cdot \left(E_{0}+o_{n}\cdot E_{0}\right)$$

Since $o_{1}=0$, this simplifies to:

$$E_{network}=E_{0}\left[\kappa_{1}+\kappa_{2}\left(1+o_{2}\right)+\kappa_{3}\left(1+o_{3}\right)+\ldots +\kappa_{n}(1+o_{n})\right]\;E_{network}=E_{0}\left(\kappa_{1}+\kappa_{2}+\kappa_{3}+\ldots +\kappa_{n}+o_{2}\kappa_{2}+o_{3}\kappa_{3}+o_{4}\kappa_{4}+\ldots +o_{n}\kappa_{n}\right)\;E_{network}=E_{0}\left(N+o_{2}\kappa_{2}+o_{3}\kappa_{3}+o_{4}\kappa_{4}+\ldots +o_{n}\kappa_{n}\right)\;E_{network}=E_{0}\left(N+N\mu_{2}o_{2}+N\mu_{3}o_{3}+N\mu_{4}o_{4}+\ldots +N\mu_{n}o_{n}\right)\;E_{network}={NE}_{0}\left(1+\mu_{2}o_{2}+\mu_{3}o_{3}+\mu_{4}o_{4}+\ldots +\mu_{n}o_{n}\right)$$

Similarly, the total network data traffic, $T_{network}$, can be written as:

$$T_{network}={N\cdot T}_{0}\cdot \left(1+\mu_{2}o_{2}+\mu_{3}o_{3}+\mu_{4}o_{4}+\ldots +\mu_{n}o_{n}\right)$$

These equations can be used to extend the principle to any level of heterogeneity for any parameter that is a function of energy.

### 3.2. Energy Model

In this study, the well-established radio-energy dissipation model, as referenced in [36,37,40,41,45,54], is utilised to calculate energy consumption and constantly update the residual energy of the network nodes. In this model, $E_{elec}$ represents the energy used by transmitter or receiver electronics in the transceiver circuit. The model accounts for power loss in both free space ($\varepsilon_{fs}$) as well as multi-path $\left(\varepsilon_{mp}\right)$ channel models. The adopted model is described as follows, adapted from [15]:

The energy spent by the radio at each node to transmit an l-bit message over a distance d is denoted as $E_{Trans}\left(l,d\right)$, where:

- l is the number of bits in the transmitted packet;
- d is the distance between the transmitter and receiver;
- $\varepsilon_{fs}$ is the free space energy dissipation coefficient;
- $\varepsilon_{mp}$ is the multi-path fading energy dissipation coefficient.

The transmission energy, $E_{Trans}\left(l,d\right)$, is calculated as:

$$E_{Trans}\left(l,d\right)=\left\{\begin{array}{c} l\times E_{elec}+l\times \varepsilon_{fs}\times d^{2};\;\;d<d_{o}\;\\ l\times E_{elec}+l\times \varepsilon_{mp}\times d^{4};\;\;d\geq d_{o} \end{array}\right.$$

where $d_{o}$ is the threshold distance that determines the switch between the free space and multi-path models and is calculated as:

do=εfsεmp

Similarly, the energy spent by the radio to receive the message is denoted by $E_{elec}\left(l\right),$ which is calculated as:

$$E_{rec}(l)=l\times E_{elec}$$

## 4. Proposed Scheme

To extend the lifetime of multi-level HWSN-based IoT networks, the primary objective of the proposed scheme is to minimise energy consumption during intra-cluster communication between the sensor nodes and cluster head. Furthermore, the scheme aims to enhance network stability in the presence of heterogeneous nodes. Here, stability is quantified by the disparity between the total number of data transmission operations accomplished before the first node failure and the total number completed before the last node failure. This can be accomplished through an appropriate cluster-head rotation algorithm that considers all the heterogeneous characteristics of nodes, ensuring minimal energy consumption and maximizing intra-cluster stability. Therefore, the cluster-head rotation that achieves stable operation while maintaining minimal intra-cluster energy consumption for a network of a multi-level heterogeneous network is discussed in following section.

### 4.1. Cluster-Head Rotation

In a sensor network composed of various types of sensor devices, even if the initial energy may be uniform, heterogeneity will naturally emerge over time. This is due to the varying energy consumption during sensing operations, which differs among sensor nodes depending on their sensing mechanisms. Consequently, cluster-head rotation methods that consider homogeneous network attributes are not practical.

**Figure 3:**
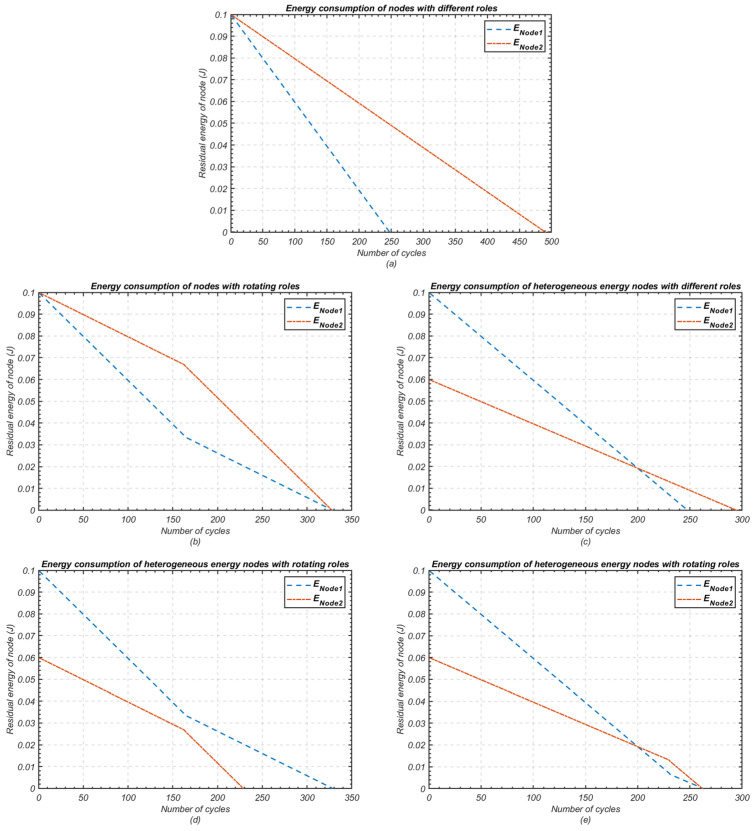
Residual energy of nodes in a WSN with varying scenarios of roles; (**a**) Homogeneous energy nodes have fixed roles; (**b**) Homogeneous energy nodes have rotating roles; (**c**) Heterogeneous energy nodes have fixed roles; (**d**) Heterogeneous energy nodes with uncoordinated role rotation; (**e**) Heterogeneous energy nodes with coordinated role rotation.

Figure 3 demonstrates the simplified problem of achieving maximum cluster lifetime in a cluster consisting of two nodes with heterogeneous parameters. The examples depicted in Figure 3a,b assume that both nodes possess identical initial energy and data rate levels (energy = 0.1 J and packet size = 4000 bits at time = 0). In Figure 3a, a static cluster-head (CH) assignment is used, with node 1 retaining the CH role throughout. Consequently, node 1 depletes its energy more quickly than node 2, leading to node 1’s shutdown after 250 cycles. It is crucial to note that time is measured in cycles, where each cycle represents a sensor’s operation, from data collection to transmission to the cluster head. Thus, in this instance, the cluster’s lifespan is 250 cycles.

In contrast, Figure 3b demonstrates that the cluster’s lifespan can be extended by rotating the CH role between the nodes. Node 1 serves as CH from cycles 0 to 164, after which node 2 takes over. Both nodes deplete their energy simultaneously after 328 cycles, thus extending the cluster lifespan to 328 cycles. It is important to highlight that the maximum lifespan occurs when both sensor nodes expire at the same time.

Figure 3c presents a scenario in which both sensor nodes have different initial energy levels (node 1: 0.1 J, node 2: 0.06 J) while maintaining the same data rate (4000 bits). However, the time gap between the failure of node 1 and node 2 does not optimise the cluster lifespan. To improve this, Figure 3d shows an applied role rotation similar to Figure 3b but with nodes of heterogeneous energy levels. The results in Figure 3d confirm that uncoordinated rotation still yields suboptimal results, emphasising the importance of coordinated role rotation.

Finally, Figure 3e shows the results of coordinated role rotation, designed to maximise cluster lifespan and minimise the time between failures of the first and second nodes. This coordinated rotation extends cluster lifespan from 250 to 262 cycles, and achieves greater stability in the network.

**Figure 4:**
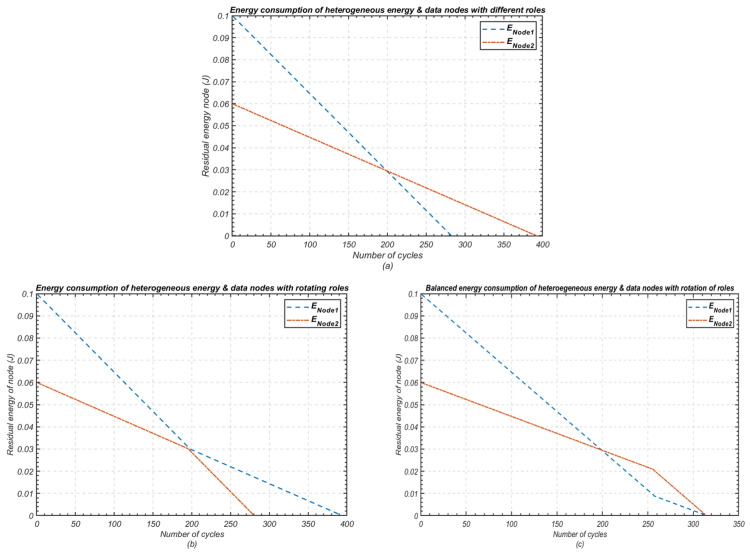
Variation in residual energy of nodes in a WSN with multi-parameter heterogeneity and role rotation; (**a**) Heterogeneous nodes with fixed roles; (**b**) Heterogeneous nodes with uncoordinated role rotation; (**c**) Heterogeneous nodes with coordinated role rotation.

Figure 4 illustrates the heightened complexity of achieving minimum energy consumption in intra-cluster communication while maintaining operational stability when nodes possess diverse initial energies and packet sizes (e.g., node 1: 5000 bits, node 2: 3000 bits). As shown in Figure 4c, coordinated role rotation yields favourable results, maximising both network lifespan and stability. However, this challenge becomes more significant in large-scale networks with multi-level and multi-parameter heterogeneity, particularly when nodes are separated by varying distances to the next hop.

**Figure 5:**
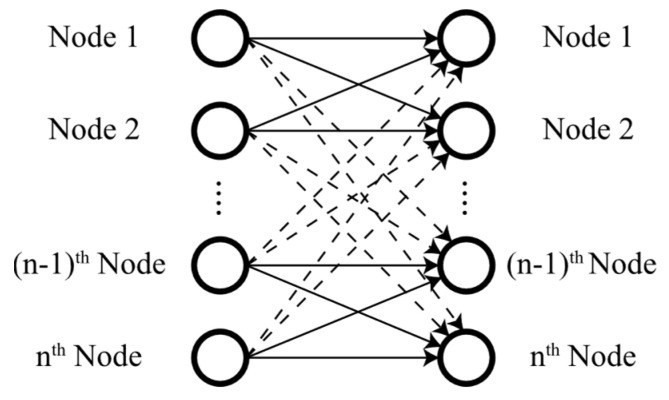
Pairwise—distance calculations between each node.

To address this, a new cluster-head rotation scheme is introduced to minimise intra-cluster energy consumption while maximising stability in heterogeneous sensor networks. Assuming the base station (BS) is aware of each node’s location, it first calculates the pairwise distances between every node within a cluster. This information is then used to implement a cluster-head rotation policy that balances energy usage and maximises network lifetime.

$$d_{ij}=\sqrt{{\left(x_{i}-x_{j}\right)}^{2}+{\left(y_{i}-y_{j}\right)}^{2}+{\left(z_{i}-z_{j}\right)}^{2}}$$

where $‘d_{ij}’$ represents the Euclidean distance of every ith node from each jth node as shown in Figure 5.

Let ‘D’ be the array containing the pairwise distances between each node in the cluster, represented as:

$$D_{\left(i,j\right)}=\left[\begin{array}{cccc} d_{11} & d_{12} & \ldots & d_{1n}\\ d_{21} & d_{22} & \ldots & d_{1n}\\ \vdots & \vdots & \ddots & \vdots \\ d_{m1} & d_{m2} & \ldots & d_{mn} \end{array}\right]$$

Based on the distance in the array ‘D’, the base station (BS) calculates the anticipated energy consumption for the transmission between each node:

$$e_{ij}=\left\{\begin{array}{c} T_{i}\cdot E_{ELEC}+T_{i}\cdot \varepsilon_{fs}{\left(d_{ij}\right)}^{2},\;d_{ij}<d_{o}\\ T_{i}\cdot E_{ELEC}+T_{i}\cdot \varepsilon_{mp}{\left(d_{ij}\right)}^{4},\;d_{ij}\geq d_{o} \end{array}\right\}$$

where $d_{o}$ is the threshold distance, calculated as:

$$d_{o}=\sqrt{\frac{\varepsilon_{fs}}{\varepsilon_{mp}}}$$

Let ‘E’ represents the array containing the anticipated energy consumption for transmission between each node pair.

$$E_{\left(i,j\right)}=\left[\begin{array}{cccc} e_{11} & e_{12} & \ldots & e_{1n}\\ e_{21} & e_{22} & \ldots & e_{1n}\\ \vdots & \vdots & \ddots & \vdots \\ e_{n1} & e_{n2} & \ldots & e_{nn} \end{array}\right]$$

Then, the total anticipated energy consumption of the nodes in a cluster while the Jth node is assumed as CH is computed in the next step.

$$E_{T}\left(j\right)=\sum_{i=1}^{n}e_{ij}$$

The average anticipated energy consumption when the Jth node is CH is given by:

$$E_{avg}(j)=\frac{\sum_{i=1}^{m}e_{ij}}{n}$$

Thus, the set of candidate cluster heads can be determined by sorting the total energy consumption values:

$$E=sort\left(E_{T}\right)=\left\{e_{1},e_{2},e_{3},\ldots ,e_{n}\right\}$$

The index of $e_{i}$ in the original unsorted set $E_{T}$ can be found as:

$$index\left(e_{i},E_{T}\right)=j$$

where:

- $e_{i}$ is an element in the sorted set E;
- $E_{T}$ is the original unsorted set;
- j represents the index of $e_{i}$ in the original unsorted set $E_{T}$.

Let E be the set of residual energies for each node:

$$E=\left\{e_{1},e_{2},e_{3},\ldots ,e_{n}\right\}$$

Thus, the index of the node with the minimum residual energy is computed as:

$${index}_{min}\left(E\right)=i$$

A node continues to act as the CH until its residual energy drops below the mean of the residual energies. The ith node is assigned as the CH:

$$A=j\left(i\right),\;\mathrm{for}\;i=1,2,3\ldots n$$

**Figure 6:**
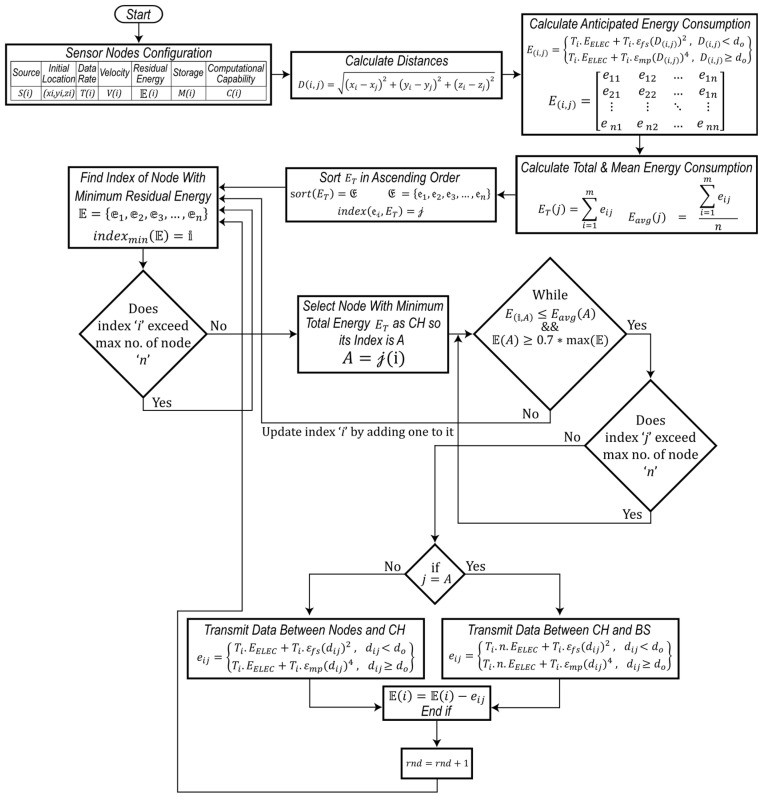
Flow chart of proposed cluster-head rotation scheme.

So, while $E_{\left(i,A\right)}\leq E_{avg}\left(A\right)\;\&\;E(A)\geq \alpha \times \max(E)$, the node with index A continues to serve as the cluster head. In this condition ‘α’ denotes the re-clustering factor and allows for tuning the frequency of re-clustering according to the required stability. Figure 6 presents the flowchart detailing the main steps in the proposed cluster-head rotation method. 

The smart sensor nodes in the WSN execute the operations outlined in Algorithms 1 and 2 at regular intervals. During typical operation, non-CH nodes conduct sensing operations and transmit their data to the CH node, as depicted in Algorithm 1.
**Algorithm 1:** Sensing nodes**Input:** Policy for varying roles 
**Output:** Data rate transmitted by each normal node in the network 
1: round = round + 1 
2: Obtain policy for the varying roles of the node over the network lifetime from BS 
3: **if** *node.role* = *Normal Node*

4:   *Perform sensing operation and detect data*

5:   **if**
*change in the parameter value,* **then**

6:       *Wake up the communication module*

7:       *send the data to the Cluster Head (CH)*

8:   **end if**

9: **elseif** *node.role = Cluster Head*

10:   *keep the communication module awake*

11:   *receive data from the member nodes*

12:   *send the aggregated data towards Base Station (BS)*
13: **end if**
**Algorithm 2:** Cluster-Head Rotation (BS)**Input:** Number of cluster member nodes ‘n’, their initial locations $\left(x_{i},y_{i},z_{i}\right)$, heterogeneous fixed data rate $T_{i}$, and initial energies $E_{i}$ of each sensor node.
**Output:** Role of each node over the network lifetime, ensuring balanced energy network operation.
1: **for** 
i←1
**to**
n
**do**
2:   **for** 
j←1
**to**
n
**do**
3:      
$D(i,j)\leftarrow \sqrt{{\left(x_{i}-x_{j}\right)}^{2}+{\left(y_{i}-y_{j}\right)}^{2}+{\left(z_{i}-z_{j}\right)}^{2}}$
4:      
$E\left(i,j\right)\leftarrow \left\{\begin{array}{c} T_{i}\cdot E_{ELEC}+T_{i}\cdot \varepsilon_{fs}\cdot {d_{ij}}^{2},\;\;\;\;\;d_{ij}<d_{o}\\ T_{i}\cdot E_{ELEC}+T_{i}\cdot \varepsilon_{mp}\cdot {d_{ij}}^{4},\;\;\;\;\;d_{ij}\geq d_{o} \end{array}\right\}$
5:   **end for**
6: **end for**
7: **for** 
j←1 
**to**
n
**do**
8:   
$E_{T}\left(j\right)\leftarrow \sum_{i=1}^{n}e_{ij}$
9:   
$E_{avg}\left(j\right)=\frac{E_{T}\left(j\right)}{n}$

10: **end for**
11: $E\leftarrow sort(E_{T})$
12: $j\leftarrow index(e_{i},E_{T})$
13: **for** rnd←1 to ${rnd}_{max}$

14:   $i\leftarrow {index}_{min}\left(E\right)$

15:   **for**
i←1 
**to**
n
**do**

16:      A←j(i)

17:      **While** $E_{\left(i,A\right)}\leq E_{avg}\left(A\right)\;\&\&\;E(A)\geq \alpha *max(E)$ **do**
18:        
  **for**
j←1 **to** n **do**

19:              **if**
j=A **then**

20:                  $e_{ij}\leftarrow \left\{\begin{array}{c} T_{i}\cdot {n\cdot E}_{ELEC}+T_{i}\cdot \varepsilon_{fs}{{(d}_{ij})}^{2},d_{ij}<d_{o}\\ T_{i}\cdot n\cdot E_{ELEC}+T_{i}\cdot \varepsilon_{mp}{{(d}_{ij})}^{4},d_{ij}\geq d_{o} \end{array}\right\}$

21:              **else**
22:                  $e_{ij}\leftarrow \left\{\begin{array}{c} T_{i}\cdot E_{ELEC}+T_{i}\cdot \varepsilon_{fs}{{(d}_{ij})}^{2},d_{ij}<d_{o}\\ T_{i}\cdot E_{ELEC}+T_{i}\cdot \varepsilon_{mp}{{(d}_{ij})}^{4},d_{ij}\geq d_{o} \end{array}\right\}$

23:                 $E\left(i\right)\leftarrow E\left(i\right)-e_{ij}$

24:              **end if**
25:        
  **end for**
26:      **break**
27:   **end for**
28:    rnd←rnd+1
29: **end for**

The role rotation policy for the nodes is established by the base station (BS) through the operations outlined in Algorithm 2. The base station establishes this policy during the network initialisation phase and communicates it to all participating nodes throughout the network’s lifetime to ensure balanced energy consumption and extended network operation.

### 4.2. Direct vs. Multi-Hop Inter-Cluster Communication

Let ‘K’ be the variable representing the possible number of hops available between the outer-most cluster head (CH) and the base station (BS). Due to unequal cluster sizes, the distances between these hops vary and are denoted as $r_{1},r_{2},r_{3,}\ldots ,r_{K}$. The energy consumed by the outermost CH in transmitting a message of size ‘l’ bits directly to the base station can be worked using the first-order radio model [15], as discussed earlier. The energy consumed during direct communication is given by:

$$E_{Direct}=E_{TX}(l,d=r_{1}+r_{2}+r_{3}\ldots +r_{K})$$

where:

$$E_{Direct}=\left\{\begin{array}{c} E_{ELEC}\cdot l+\epsilon_{fs}\cdot l\cdot d^{2}\;if\;d\leq d_{o}\\ E_{ELEC}\cdot l+\epsilon_{mp}\cdot l\cdot d^{4}\;otherwise \end{array}\right\}$$

Similarly, energy consumed during multi-hop inter-cluster communication can be computed as:

$$E_{Multihop}=K*E_{TX}\left(l,d={r_{1}}^{2}+{r_{2}}^{2}+\ldots +{r_{K}}^{2}\right)+(K-1)E_{RX}\;E_{Multihop}=K\left[E_{ELEC}*l+\epsilon_{fs}*l*\left({r_{1}}^{2}+{r_{2}}^{2}+\ldots +{r_{K}}^{2}\right)\right]+(K-1)\left[E_{ELEC}*l\right]$$

To determine when inter-cluster multi-hop routing is required, the following two cases are considered.

Case 1: $d\leq d_{o}$

If the overall distance ‘d’ between a cluster head and the base station is less than or equal to the threshold distance ‘$d_{o}$’ as defined by the radio model, then energy consumed by direct communication is:

$$E_{Direct}=E_{ELEC}*l+\epsilon_{fs}*l*d^{2}$$

Direct communication is preferred if the following condition is satisfied:

$$E_{Direct}<E_{Multihop}\;E_{ELEC}*l+\epsilon_{fs}*l*d^{2}<l\left[\left[KE_{ELEC}+{K\epsilon }_{fs}\left({r_{1}}^{2}+{r_{2}}^{2}+\ldots +{r_{K}}^{2}\right)\right]+\left[{KE}_{ELEC}-E_{ELEC}\right]\right]\;d^{2}<2\frac{E_{ELEC}\left(K-1\right)}{\epsilon_{fs}}+K\left({r_{1}}^{2}+{r_{2}}^{2}+\ldots +{r_{K}}^{2}\right)$$

Case 2: $d>d_{o}$

If the overall distance between the cluster head and the base station exceeds the threshold distance ‘$d_{o}$’, the energy consumed by direct communication is computed as:

$$E_{Direct}=E_{ELEC}*l+\epsilon_{mp}*l*d^{4}$$

Direct communication is preferred if:

$$E_{Direct}<E_{Multihop}\;E_{ELEC}*l+\epsilon_{mp}*l*d^{4}<l\left[\left[KE_{ELEC}+{K\epsilon }_{fs}\left({r_{1}}^{2}+{r_{2}}^{2}+\ldots +{r_{K}}^{2}\right)\right]+\left[{KE}_{ELEC}-E_{ELEC}\right]\right]\;d^{4}<2\left(K-1\right)\frac{E_{ELEC}}{\epsilon_{mp}}+K({r_{1}}^{2}+{r_{2}}^{2}+\ldots +{r_{K}}^{2})\frac{\epsilon_{fs}}{\epsilon_{mp}}$$

#### Energy Balanced Inter-Cluster Multi-Hop Communication

**Figure 7:**
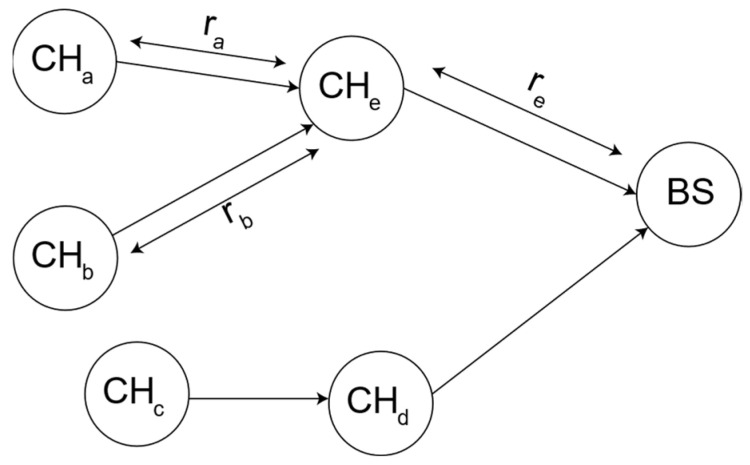
Multi-hop inter-cluster routing to transmit data to BS.

If the network size and configuration favour multi-hop routing as the more energy-efficient option, an issue of imbalance load among cluster heads may arise, as illustrated in Figure 7. Cluster heads that are located closer to the base station (BS) experience a heavier relaying load, particularly in large-scale networks, as they are responsible for forwarding data from distant cluster heads to the BS. This uneven energy consumption can lead to premature failure of the nearer cluster heads, reducing the overall network lifespan.

The proposed scheme explores the condition to balance the load among cluster heads, thereby increasing network lifetime and stability. The following mathematical model illustrates this:

Let the cluster heads ${CH}_{a}$ and ${CH}_{b}$ be located at the same level and have the same number of nodes ‘a’, and the same data rate on them, denoted by ‘l’.

The total energy consumption for ${CH}_{a}$ is expressed as:

$$E_{a-TOTAL}=a\cdot l\cdot E_{ELEC}+EDA+l{\cdot E}_{ELEC}+l\cdot \epsilon_{fs}\cdot {r_{a}}^{2}$$

Assuming that the energy consumption for ${CH}_{a}$ and ${CH}_{b}$ is equal, then

$$E_{a-TOTAL}=E_{b-TOTAL}$$

Let ‘e’ be the number of nodes in cluster ${CH}_{e}$. The total energy consumption of ${CH}_{e}$ is given by:

$$E_{e-TOTAL}=\left(a+b\right)\cdot l\cdot E_{ELEC}+{e\cdot l\cdot E}_{ELEC}+EDA+{l\cdot E}_{ELEC}+l{\cdot \epsilon }_{fs}\cdot {r_{e}}^{2}$$

The condition for balanced energy routing implies:

$$E_{a-TOTAL}=E_{e-TOTAL}\;a\cdot l\cdot E_{ELEC}+EDA+l\cdot E_{ELEC}+l\cdot \epsilon_{fs}\cdot {r_{a}}^{2}=\left(a+b\right)\cdot l\cdot E_{ELEC}+{e\cdot l\cdot E}_{ELEC}+EDA+{l\cdot E}_{ELEC}+l\cdot \epsilon_{fs}\cdot {r_{e}}^{2}$$

Given that a=b≠e, the condition can be simplified as:

$$a{\cdot E}_{ELEC}+\epsilon_{fs}\cdot {r_{a}}^{2}=2\cdot a\cdot E_{ELEC}+{e\cdot E}_{ELEC}+\epsilon_{fs}\cdot {r_{e}}^{2}\;\epsilon_{fs}\cdot {r_{a}}^{2}=\left(a+e\right)\cdot E_{ELEC}+\epsilon_{fs}{\cdot r_{e}}^{2}\;{r_{a}}^{2}=\frac{{\left(a+e\right)\cdot E}_{ELEC}}{\epsilon_{fs}}+{r_{e}}^{2}$$

This equation determines the condition for balanced energy routing across multiple cluster heads in a multi-hop scenario.

### 4.3. Relay-Node Selection and Rotation

To improve the network lifetime while maintaining balanced energy consumption among network nodes, relay nodes in addition to cluster heads are utilised in the proposed method. The scheme is adaptable to varying network scales, determining the most energy-efficient location for relay nodes. Given that the ith cluster head ${CH}_{i}$ needs to transmit data to the destination (i.e., base station BS), direct transmission becomes energy efficient if the condition $d\leq d_{0}^{min}$ is satisfied. The value of $d_{0}^{min}$ can be calculated as:

$$d_{0}^{min}=\sqrt[{\gamma }]{\frac{\varepsilon_{o}(\gamma )}{1-2^{-\frac{\gamma }{2}}}}$$

**Figure 8:**
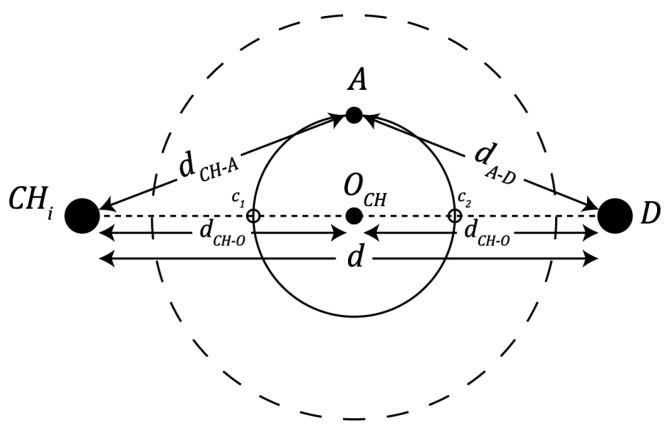
Relay-node search region based on energy change.

Figure 8 illustrates the search region for relay nodes in the network. The choice of the relay node is determined by considering the distance between the cluster head ${CH}_{i}$ and the destination node D, aiming to reduce energy consumption while maintaining efficient network performance.

In the diagram, $d_{CH-O}$ represents the distance between the cluster head ${CH}_{i}$ and the central point O, which is a potential relay position. Distances $d_{CH-A}$ and $d_{A-D}$ denote the distances between the cluster head and a potential relay node A, and between the relay node and the destination D, respectively. The relay-node search area, represented by the dashed circle, is governed by the energy dynamics of the network, with the goal of balancing the load across relay nodes to optimise energy efficiency.

Parameter γ can take values of 2 or 4, based on the first-order radio model parameters $E_{ELEC}$, $\varepsilon_{fs},$ and $\varepsilon_{mp}$, which influence the calculation of the threshold distances. The parameters are defined as follows:

- For γ=2, the energy threshold is given by $\varepsilon_{o}\left(\gamma \right)=\frac{E_{ELEC}}{\varepsilon_{fs}}$, reflecting the free space model.
- For γ=4, the energy threshold is $\varepsilon_{o}\left(\gamma \right)=\frac{E_{ELEC}}{\varepsilon_{mp}}$, representing the multi-path fading model.

These energy thresholds dictate the conditions under which direct or multi-hop communication is more energy efficient. When γ=2, the network operates under the free-space propagation model, and when γ=4, the multi-path fading model is applied. The selection of relay nodes is thus guided by these parameters, ensuring minimal energy consumption and maximising the network’s lifetime.

**Figure 9:**
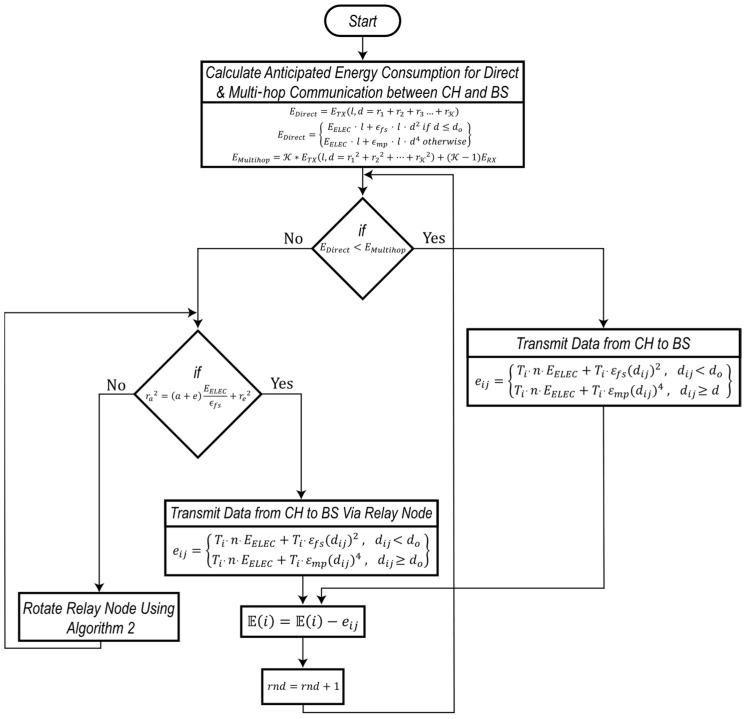
Flow chart of the proposed relay selection and rotation method.

Figure 9 presents the flow chart detailing the stepwise operation of the proposed balanced energy and maximum network lifetime inter-cluster multi-hop communication method. The process begins with the farthest cluster head from the base station and calculates the number of hops required for energy-efficient transmission. This approach continues progressively towards relay nodes closer to the base station.

The goal is to identify the optimal relay-node positions to ensure that direct transmission from the closer relay nodes is more energy efficient than multi-hop routing from farther nodes. This strategy extends the scalability of the network and allows the proposed method to operate adaptively in networks of varying scale, which is essential for supporting IoT infrastructure. By continuously adjusting based on the energy consumption model, the method helps balance the energy usage across the network and prolong its overall lifetime.

The pseudo-code in the Algorithm 3 ensures the energy efficient relay-node selection and rotation of relay role among nodes within the set of candidate relay nodes, all executed at an appropriate frequency to optimise energy usage and network stability.
**Algorithm 3:** Relay-Node Selection & Rotation Policy**Input:** ‘n’ number nodes, their locations $\left(x_{i},y_{i},z_{i}\right)$, heterogeneous fixed data rate $T_{i}$, and initial energies $E_{i}$ of each sensor node, sensing region and its boundaries. Total number of cluster heads ρ and total number of sensor nodes N.
**Output:** A policy for the selection of relay node for each cluster head to transmit its cluster data to the base station in an energy efficient and stable manner. Provides balanced energy transmission between nodes through timely and energy efficient rotation of relay nodes.
1: $d_{0}^{min}\leftarrow \sqrt[{\alpha }]{\frac{\varepsilon_{o}\left(\alpha \right)}{1-2^{-\frac{\alpha }{2}}}}$

2: **for**
i=1 **to** ρ **do**

3:   $d_{{CH}_{i}-BS}\leftarrow d$
4:   **if**
$d_{{CH}_{i}-BS}>d_{0}^{min}$ **then**
5:       *Compute the radius* ‘A’
6:       **for**
j=1 **to** N **do**
7:           **if**
$d_{ij}<A$ **then**
8:               $R_{i-candidate}\leftarrow j$
9:               **While**
$E_{\left({CH}_{i},j\right)}\leq E_{avg}\left(j\right)\;\&\&\;E(j)\geq \alpha *max(E)$ **do**
10:                   *Continue to transmit data* via *relay node* ‘j’
11:                   $E\left(j\right)\leftarrow E\left(j\right)-e_{j-BS}$
12:              **else**

13:                   *Rotate relay role to next candidate*
14:              **Break**

15:          **end if**
16:      **end for**
17:   **else**
18:      *Transmit data* via *direct communication between* ‘${CH}_{i}$’ *and* ‘BS’ 
19:      $E_{Direct}=\left\{\begin{array}{c} E_{ELEC}*l+\epsilon_{fs}*l*d^{2}\;if\;d\leq d_{o}\\ E_{ELEC}*l+\epsilon_{mp}*l*d^{4}\;otherwise \end{array}\right\}$

20:      $E\left(i\right)\leftarrow E\left(i\right)-E_{Direct}$
21:   **end if**
22:   rnd←rnd+1

23: **end for**

## 5. Results and Discussion

This section presents the evaluation of the proposed techniques using MATLAB R2022a simulations, chosen for its ability to accurately model the complex dynamics of energy-efficient communication in heterogeneous WSNs and IoT environments. MATLAB provides robust numerical computation capabilities, which are essential for implementing the proposed cluster-head and relay-node rotation algorithms in a precise and controlled manner. Moreover, MATLAB’s advanced visualisation tools offer a clear means for comparing the performance of different quality of service (QoS) metrics, such as energy consumption, network lifetime, and throughput—metrics that are crucial for assessing the effectiveness of the proposed methods.

The choice of MATLAB as the simulation environment is not arbitrary but is instead keeping in view its wide use for evaluating state-of-the-art protocols, such as RLEACH, CRPFCM, and EERPMS. This consistency across studies facilitated a fair and reliable comparison between the proposed methods and existing techniques, ensuring that the novel contributions of this research are accurately evaluated against established approaches.

The evaluation metrics used in this study, i.e., energy consumption, network lifetime, and throughput—are central to the performance assessment of WSN-based IoT systems, particularly in the context of heterogeneous sensor networks. These metrics were chosen due to their direct correlation with the main objectives of the study, i.e., improving energy efficiency, extending the operational lifespan of the network, and maximising the amount of data successfully transmitted to the base station.

### 5.1. Intra-Cluster Communication

The simulation parameters employed throughout the experiments to evaluate the performance of the proposed cluster-head rotation method are summarised in Table 1. The proposed method is first tested using clusters composed of three and five nodes, each with heterogeneous initial energy levels ranging from 0.05 J to 0.2 J, ensuring that the total energy of nodes within each cluster is 0.5 J. Additionally, the nodes are assumed to generate data packets of varying sizes within the range of 1000 bits to 8000 bits. The proposed heterogeneity model ensures that the total data transmitted in one cycle by all nodes in a cluster equals 20,000 bits.

#### 5.1.1. Comparison of Residual Energy

**Figure 10:**
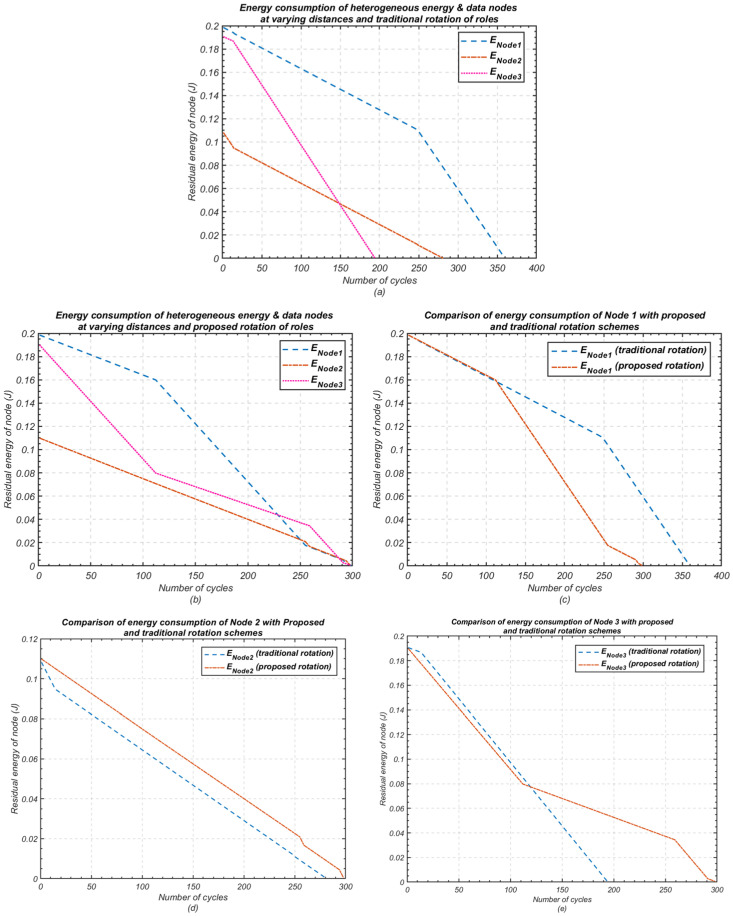
Residual energy using the proposed cluster-head rotation scheme compared with the traditional method (*n* = 3); (**a**) Traditional rotation or node roles; (**b**) Proposed rotation of node roles; (**c**) Comparison of energy consumption of node 1; (**d**) Comparison of energy consumption of node 2; (**e**) Comparison of energy consumption of node 3.

In this experiment, the residual energy of each node was compared to assess the energy efficiency of the proposed intra-cluster communication scheme, which utilises a cluster-head rotation approach. Figure 10 illustrates the comparison of the residual energies of nodes during intra-cluster communication, contrasting the proposed rotation method (Figure 10a) with the traditional rotation method (Figure 10b) for a cluster consisting of three nodes.

The traditional rotation method demonstrates a rapid depletion of energy, with the first node in the cluster exhausting its energy after 194 cycles of data transmission. The last node in the cluster depletes its energy after 360 cycles. This disparity in energy depletion imposes limitations on the cluster’s lifetime and coverage, with a notable impact on first node death scale, thereby reducing stability.

In contrast, the proposed cluster-head rotation scheme significantly extends the lifespan of the network. The first node’s energy depletion is delayed until 298 cycles, with the nodes exhibiting converging behaviour in their energy consumption. This convergence indicates improved stability and more efficient utilisation of available energy, thereby prolonging the network’s operation.

**Figure 11:**
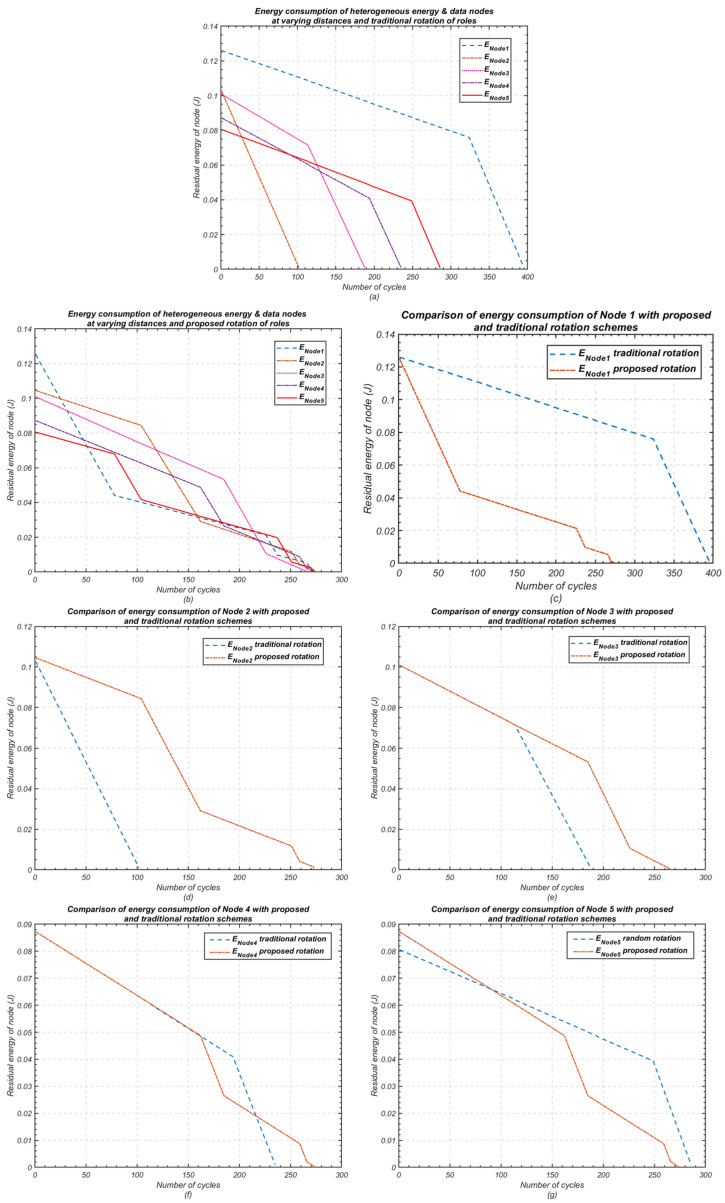
Residual energy using the proposed cluster-head rotation scheme compared with the traditional rotation (*n* = 5); (**a**) Traditional rotation of node roles; (**b**) Proposed rotation of node roles; (**c**) Comparison of energy consumption of node 1; (**d**) Comparison of energy consumption of node 2; (**e**) Comparison of energy consumption of node 3; (**f**) Comparison of energy consumption of node 4; (**g**) Comparison of energy consumption of node 4.

Figure 11 presents results consistent with those observed in Figure 10, demonstrating the effectiveness of the proposed cluster-head rotation scheme as the number of nodes in the cluster increases to five. These results validate the scalability of the proposed method, confirming that it can efficiently manage energy consumption across larger clusters while maintaining balanced energy utilisation and extended network lifetime.

The experiments conducted with clusters of nodes *n* = 3 and *n* = 5 were specifically chosen to illustrate the scalable and adaptable behaviour of the proposed cluster-head rotation method across different network sizes. These two configurations effectively represent small and moderately sized clusters, which are typical in many HWSN-based IoT applications. By focusing on these scenarios, the experiments provide a clear demonstration of the method’s capacity to manage energy efficiently and extend network lifetime within smaller-scale deployments, where resource constraints are critical.

The choice of *n* = 3 and *n* = 5 is sufficient for assessing the proposed method’s performance because they offer a simplified testing ground. These cluster sizes allow for detailed observation of energy consumption patterns and the benefits of the proposed rotation scheme in a controlled environment. The results show significant improvements in energy management, as seen through the extended network lifespan and balanced energy utilisation, even as the cluster size increases from three to five nodes. It is clear that these results can easily be extended to other cluster sizes.

Additionally, these experiments serve as an initial demonstration of the proposed method’s scalability. The consistent outcomes across both configurations suggest that the proposed approach can maintain energy efficiency and stability, even as the network scales up. This lays the groundwork for the full-scale network simulations presented in subsequent sections, where larger and more complex network topology of full networks is examined. By first testing on smaller clusters, the experiments provide foundational evidence of the method’s adaptability, ensuring that the insights gained here translate into larger network scenarios.

Thus, the use of *n* = 3 and *n* = 5 clusters is not only adequate but also effective. It offers a clear, focused analysis of the proposed method before introducing the complexities of larger network simulations. These smaller-scale experiments provide crucial validation of the method’s core principles, which are further substantiated when applied to larger networks later in the study.

**Figure 12:**
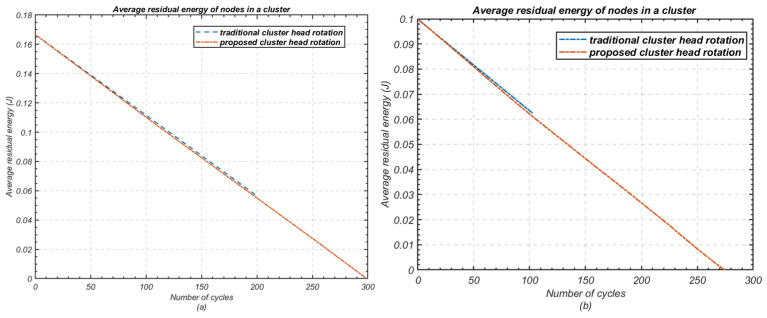
Comparison of average residual energy of nodes in a cluster. (**a**) Number of nodes *n* = 3; (**b**) Number of nodes *n* = 5.

**Figure 13:**
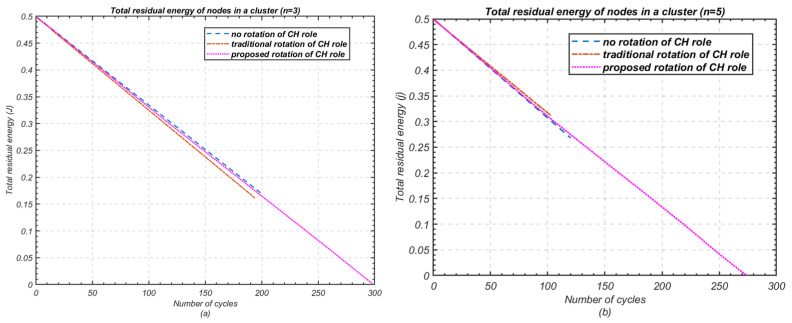
Comparison of total remaining energy of nodes in a cluster; (**a**) Number of nodes *n* = 3; (**b**) Number of nodes *n* = 5.

To compare the unused energy of nodes before the cluster reaches the end full effectiveness (i.e., at least one node is dead), a comparison of the average and total remaining energy of the cluster during each cycle of data transmission before the death of the first node is presented in Figure 12 and Figure 13, respectively, for both scenarios, with *n* = 3 and *n* = 5. The results confirm that 33.47% of the total initial energy is still unused in a cluster of three nodes before the cluster becomes ineffective with the death of the first node in the 198*th* cycle. Similarly, 62.30% of the average residual energy is unused in a cluster of five nodes before the death of first node in the 103rd cycle. 

#### 5.1.2. Average Energy Consumption

**Figure 14:**
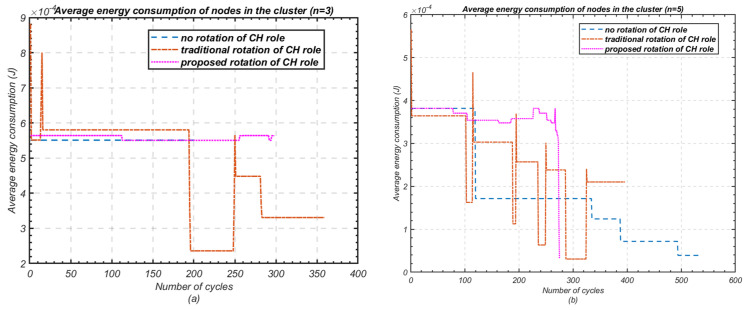
Average energy consumption of nodes within a cluster; (**a**) Number of nodes *n* = 3; (**b**) Number of nodes *n* = 5.

Average energy consumption of nodes within a cluster is an important criterion in terms of stable operation of the cluster. To evaluate this, the proposed cluster-head rotation scheme was compared against both the traditional rotation scheme and the method with no cluster-head role rotation. As shown in Figure 14, the proposed rotation scheme ensures relatively stable energy consumption, effectively utilising the available energy throughout the cluster’s operation.

In contrast, the traditional rotation scheme results in fluctuating energy consumption values, due to early node failures and demonstrating its inefficiency in managing the energy consumption balance. On the other hand, the fixed cluster-head method maintains a steady energy consumption rate until the first node dies, at which point the cluster’s overall energy efficiency drops. Notably, both the traditional rotation and fixed cluster-head methods continue to operate even after the first node’s death, leading to a complete waste of energy since no further throughput is transmitted to the base station.

#### 5.1.3. Throughput to the BS

**Figure 15:**
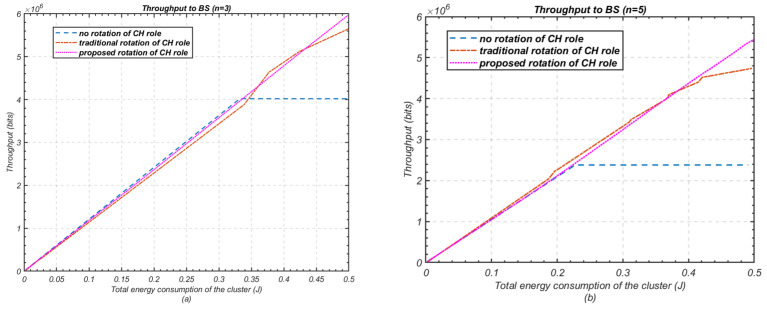
Performance comparison in terms of throughput to BS against energy consumed; (**a**) Number of nodes *n* = 3; (**b**) Number of nodes *n* = 5.

The effectiveness of a heterogeneous WSN-based IoT system is evaluated by the amount of data collected and delivered to the base station. Figure 15 compares the throughput achieved in various cluster-head rotation schemes, for clusters with both three and five nodes.

**Figure 16:**
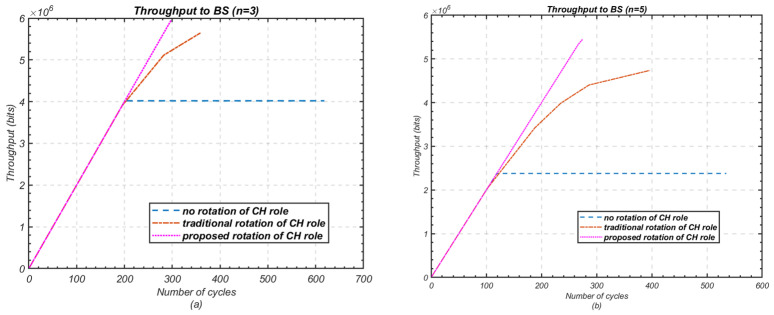
Performance comparison in terms of throughput to BS against the network lifetime; (**a**) Number of nodes *n* = 3; (**b**) Number of nodes *n* = 5.

The results in Figure 15a show that the proposed cluster-head rotation method significantly outperforms both the fixed cluster-head and traditional cluster-head rotation schemes. Specifically, it delivers a 50% increase in throughput compared to the fixed cluster-head scheme and a 7.1% increase over the traditional cluster-head rotation method when *n* = 3. For *n* = 5, the proposed method achieves an even more substantial improvement, yielding a 125% and 13% increase in throughput compared to the fixed and traditional rotation methods, respectively as shown in Figure 16.

These results clearly demonstrate the superiority of the proposed cluster-head rotation scheme in terms of data transmission efficiency to the base station.

#### 5.1.4. Network Lifetime Achieved by Intra-Cluster Communication

**Figure 17:**
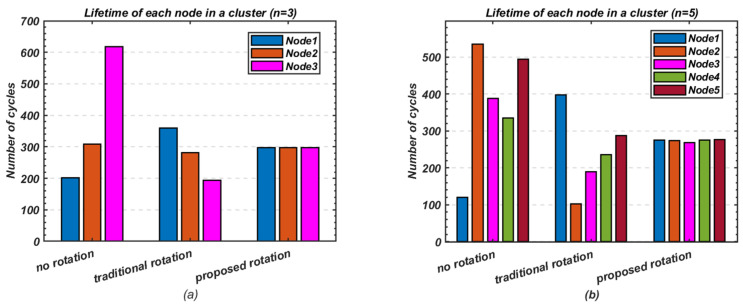
Comparison of lifetime and stability; (**a**) Number of nodes *n* = 3; (**b**) Number of nodes *n* = 5.

This experiment explores the efficient utilisation of heterogeneous node resources in small-scale networks, where multi-hop routing may not be necessary for communication between cluster heads and the base station. Figure 17 highlights the performance of the proposed method in extending the lifetime of each node within a cluster compared to the fixed cluster-head and traditional cluster-head rotation schemes.

Without any rotation, the difference between the death of the first and last node is the most noticeable, with significant variations between nodes. However, this difference is reduced with the traditional rotation method and further minimised with the proposed rotation scheme. For *n* = 3, the difference between the first and last node’s death decreases from 417 cycles to 165 cycles when using traditional rotation, while the proposed method achieves a near-equal node lifespan. For *n* = 5, the difference in node death reduces from 415 cycles to 294 cycles with traditional rotation, and the proposed method narrows this further, with only 8 cycles difference between the first and last node’s death.

**Figure 18:**
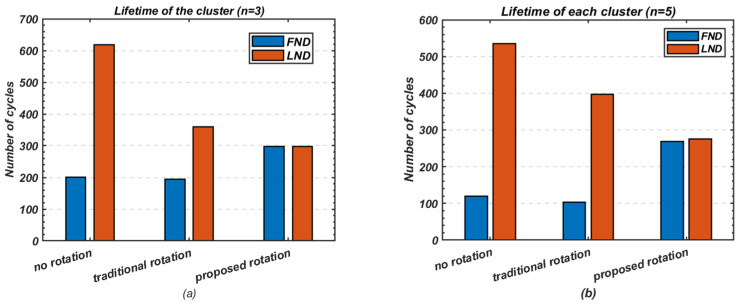
Comparison of overall cluster lifetime on (FND) and (LND) scales; (**a**) Number of nodes *n* = 3; (**b**) Number of nodes *n* = 5.

Figure 18 summarises the overall cluster lifetime, comparing the first node death (FND) and last node death (LND) across the different schemes. The proposed cluster-head rotation method notably enhances the cluster’s lifetime on the FND scale while also maintaining stable performance. This makes it particularly suitable for scenarios requiring consistent and reliable operation, ensuring the cluster operates efficiently even as nodes approach their energy depletion. 

### 5.2. Inter-Cluster Communication

The performance of the proposed inter-cluster communication method, based on relay-node selection and rotation, was evaluated using the parameters shown in Table 2. The network was tested with 100 nodes uniformly randomly distributed, each having heterogeneous initial energy ranging from 0.5 J to 2 J, with the total energy of the network set at 100 J. Similarly, nodes generated data packets ranging from 1000 bits to 4000 bits, maintaining a total of 200,000 bits transmitted across the network.

#### 5.2.1. Network Lifetime and Remaining Energy of Each Node

**Figure 19:**
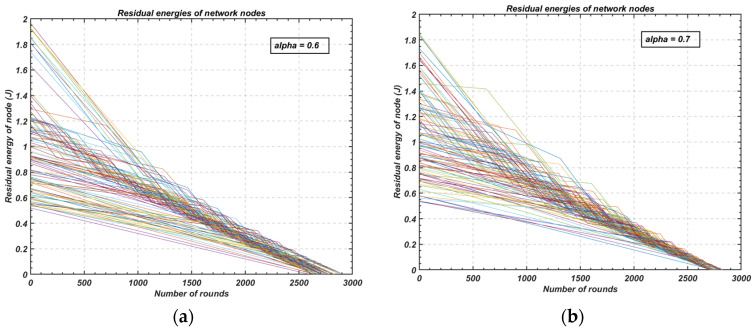
Comparison of residual energy of nodes with the proposed relay-node rotation scheme: (**a**) alpha = 0.6; (**b**) alpha = 0.7.

The proposed relay-node rotation method has been evaluated for its impact on network lifetime, specifically considering the variable initial energy levels of the nodes. Figure 19 demonstrates the residual energy of each node after each round of operation. The term ‘round’ refers to a complete cycle of sensing and data transmission to the cluster head, followed by data transmission from the cluster head to the base station via multi-hop communication.

In Figure 19a, for an alpha value of 0.6, the proposed relay-node rotation scheme achieves an intra-cluster energy balance. Despite this, the first node dies after 2602 rounds, while the last node dies at 2890 rounds. When the alpha value is increased to 0.7 (Figure 19b), there is a noticeable improvement in the network’s stability and convergence of node lifetimes. Specifically, the first node death (FND) is delayed to the 2703rd round, while the last node death (LND) occurs at the 2830th round. These results illustrate the flexibility of the proposed scheme in adjusting the frequency of relay-node rotation based on network requirements, leading to enhanced energy balance and prolonged network operation.

### 5.3. Overall Performance Evaluation

This section evaluates the overall effectiveness of the proposed cluster-head rotation and balanced inter-cluster communication methods. Performance is assessed in terms of residual energy, network lifetime, and throughput delivered to the base station with state-of-the-art methods, i.e., RLEACH [55], CRPFCM [56,57], and EERPMS [57].

#### 5.3.1. Overall Residual Energy of the Network

**Figure 20:**
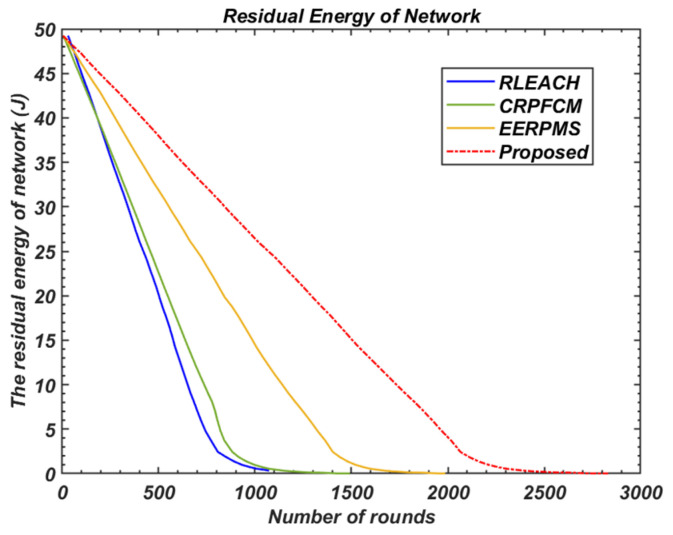
Comparison of residual energy of network.

As shown in Figure 20, the proposed method significantly outperforms other methods in terms of residual energy. This is primarily due to the careful selection of relay nodes and the minimisation of energy consumption during inter-cluster communication. In particular, the proposed method maintains higher residual energy compared to RLEACH, CRPFCM, and EERPMS, thereby ensuring prolonged network effectiveness.

In heterogeneous sensor networks, cluster heads consume significantly more energy than normal nodes due to additional responsibilities such as data aggregation and relaying. As a result, they deplete their energy more quickly. However, by considering nodes with greater resources for relaying operations and minimizing overall energy consumption, the proposed method efficiently balances the network load and extends the operational lifetime of the network.

#### 5.3.2. Overall Network Lifetime

**Figure 21:**
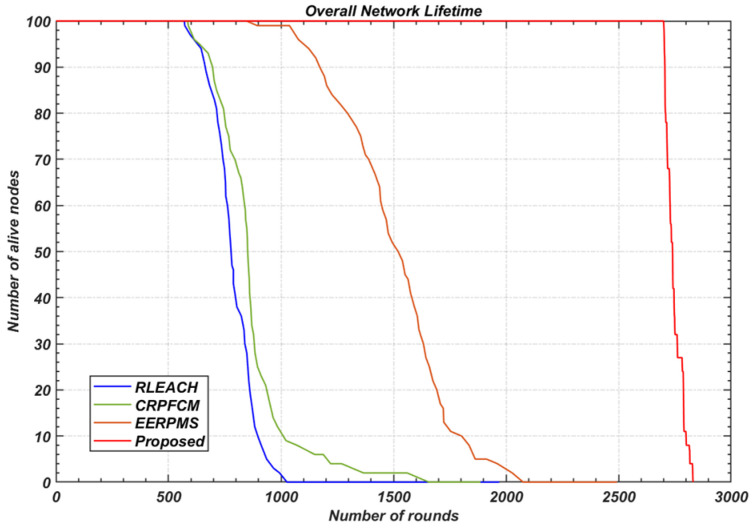
Number of live nodes vs. number of rounds.

**Figure 22:**
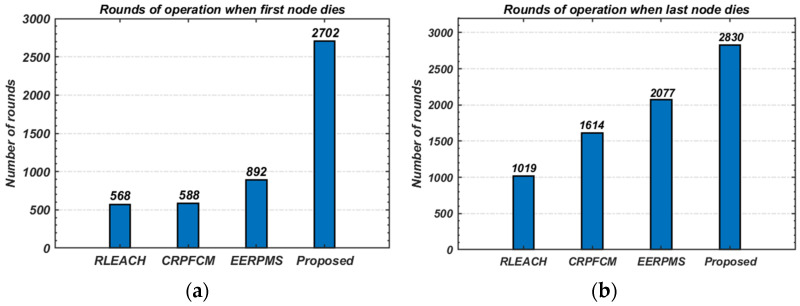
Comparison of overall network lifetime: (**a**) FND scale; (**b**) LND scale.

Network lifetime is a critical performance metric, often measured in terms of the number of rounds until the first node dies (FND), half the nodes die (HND), or the last node dies (LND). Figure 21 illustrates the survival rate of nodes over the network’s operational period. The proposed method significantly extends the network lifetime on both the FND and LND scales, as shown in Figure 22.

Specifically, the proposed method increases the number of rounds before the first node dies (FND) to 2702, compared to just 568 rounds for RLEACH, 588 rounds for CRPFCM, and 892 rounds for EERPMS (Figure 22a). Similarly, the proposed method extends the network lifetime on the LND scale to 2830 rounds, compared to 1019 rounds for RLEACH, 1614 rounds for CRPFCM, and 2077 rounds for EERPMS (Figure 22b).

This significant improvement in network lifetime can be attributed to the proposed method’s ability to maintain energy balance across the network, particularly by evenly distributing energy-intensive tasks such as data aggregation and relaying across nodes with higher residual energy.

#### 5.3.3. Throughput to Base Station

In the context of heterogeneous wireless sensor networks (HWSNs) deployed within IoT systems, throughput is a crucial performance indicator. It represents the total volume of data successfully gathered by the sensor nodes and transmitted to the base station [58]. Given the integrity of data collected and transmission in IoT applications, evaluating throughput provides valuable insight into the overall efficiency of energy-conscious routing protocols, which are integral to maintaining network longevity and reliability.

**Figure 23:**
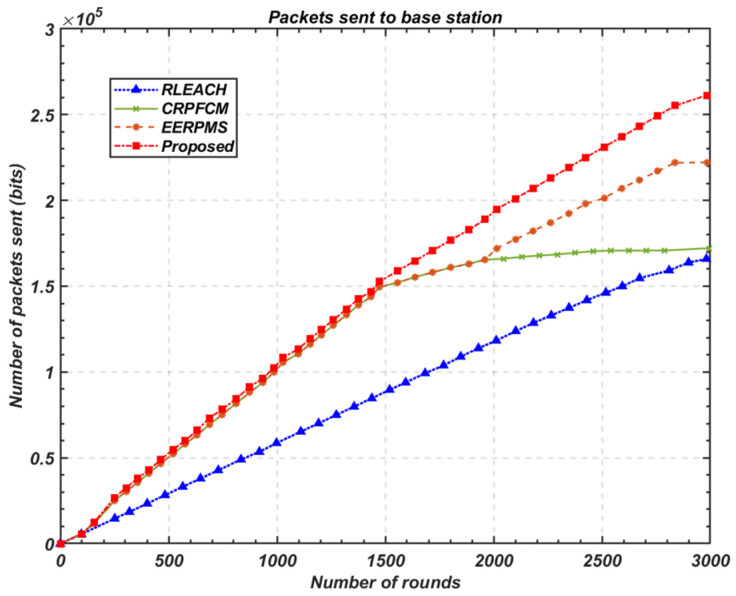
Comparison of throughput to base station.

Figure 23 illustrates the comparison between the proposed balanced-energy routing scheme, which incorporates both cluster-head rotation and relay-node rotation, and state-of-the-art protocols such as RLEACH [55], CRPFCM [56,57], and EERPMS [57]. The results demonstrate that the proposed method achieves a significant increase in throughput, outperforming its peers across all tested scenarios. Specifically, the proposed method shows a 17.63% higher data packet delivery rate compared to EERPMS [57], a protocol designed for energy efficiency and routing in heterogeneous networks. This substantial improvement can be attributed to the more dynamic role-rotation strategies employed by the proposed method, which ensure that cluster heads and relay nodes are selected based not only on their residual energy but also on their location and data generation characteristics, leading to optimal energy utilization.

Moreover, when comparing the proposed method with CRPFCM [56,57] and RLEACH [55], the throughput increase is even more noticeable. The proposed method delivers 51.75% more data packets than CRPFCM and 57.44% more than RLEACH. These enhancements can be critically examined in light of the shortcomings in the competing protocols. RLEACH, for instance, primarily focuses on random and periodic rotation of cluster heads without sufficient consideration of heterogeneity in node energy levels or traffic loads, which often leads to uneven energy depletion and reduced network effectiveness. CRPFCM, while incorporating fuzzy clustering techniques, lacks the fine-tuned balance between intra-cluster and inter-cluster communication, particularly in large-scale heterogeneous networks. This limitation results in suboptimal data transmission rates and premature exhaustion of energy in certain nodes, particularly cluster heads.

The significant improvement in throughput achieved by the proposed method underscores the importance of a multi-layered approach to energy management in HWSNs. By balancing energy consumption not only within clusters but also across clusters through relay-node rotation, the method ensures that the network remains operational for longer periods, allowing more data to be transmitted to the base station before nodes start to fail. Furthermore, the incorporation of flexible relay-node selection criteria, based on both distance and energy considerations, minimises the energy overhead associated with multi-hop communication, further contributing to the enhanced throughput.

These results highlight the efficacy of the proposed method in addressing the key challenges of energy efficiency and data transmission in heterogeneous IoT networks. The ability to deliver significantly more data packets while maintaining balanced energy consumption across the network is a critical advantage, particularly in scenarios where large-scale data collection and long network lifetimes are paramount.

## 6. Conclusions and Future Scope

In this paper, cluster-head and relay-node rotation-based balanced energy routing for the IoT-based HWSNs is proposed. The proposed cluster-head rotation method addresses the challenges of energy balance and extended network lifetime in intra-cluster communication, even in scenarios involving multi-parameter and multi-level heterogeneity. The simulation results validate consistent performance by the cluster-head rotation method for n-level of heterogeneity according to proposed multi-level heterogeneity model.

Additionally, a condition between direct and multi-hop inter-cluster communication is determined and a method proposed to compute the optimum region for the choice of next-hop relay in the case when multi-hop inter-cluster communication is energy efficient. A novel balanced energy relay-node rotation algorithm is also introduced to ensure energy-efficient operation in inter-cluster communication, thereby extending both network lifetime and throughput.

The performance of the proposed cluster-head rotation is compared with fixed cluster-head as well as traditional cluster-head rotation schemes that do not account for appropriate exploitation of the heterogeneity factor and the minimum energy consumption rate. The proposed cluster-head rotation method demonstrates effective utilisation of available energy with increased throughput and enhanced network lifetime of the nodes in a cluster. Moreover, the optimum choice of relay-node and periodic rotation of the relay role among nodes in the given energy-efficient region extends overall network lifetime, stability, energy efficiency, and throughput, as compared to RLEACH, CRPFCM, and EERPMS.

The use of mobile nodes has recently been introduced in IoT-based sensor networks to further their applications. This introduces the challenges of robust and reliable connectivity and therefore data exchange. Thus, we intend to investigate the ways in which more real-life scenarios of mobility can be introduced into IoT-based heterogeneous wireless sensor networks.

## Figures and Tables

**Table 1:** Simulation parameters.

Parameter	Value
Number of nodes (*n*)	3 and 5
Range of heterogeneous initial energies of nodes	(0.05−0.2) J
Total energy of nodes in a cluster	0.5 J
Range of heterogeneous packet size (*l*)	(1000−8000) bits
Total data transmitted by nodes in a cluster	20,000 bits
Electronic circuitry ($E_{elec}$)	50 nJ/bit/m^2^
Amplifier energy for free space ($\varepsilon_{fs}$)	10 pJ/bit/m^2^
Amplifier energy for multipath ($\varepsilon_{mp}$)	pJ/bit/m^4^

**Table 2:** Simulation parameters for inter-cluster communication.

Parameter	Value
Number of nodes (*N*)	100
Range of heterogeneous initial energies of nodes	(0.5−2) J
Total energy of nodes in the network	100 J
Range of heterogeneous packet size (*l*)	(1000−4000) bits
Total data transmitted by nodes in a transmission	200,000 bits
Electronic circuitry ($E_{elec}$)	50 nJ/bit/m^2^
Amplifier energy for free space ($\varepsilon_{fs}$)	10 pJ/bit/m^2^
Amplifier energy for multipath ($\varepsilon_{mp}$)	0.14 pJ/bit/m^4^

## Data Availability

There are no data to be made available.
